# 3D & 4D Printing—In Engineering Applications

**DOI:** 10.3390/ma19071307

**Published:** 2026-03-26

**Authors:** Tomasz Kozior, Jerzy Bochnia

**Affiliations:** Faculty of Mechatronics and Mechanical Engineering, Kielce University of Technology, 25-314 Kielce, Poland

## 1. State of the Art

An analysis of the current state of the development of 3D and 4D printing technology indicates a focus on two basic trends: rapid product development and industrial manufacturing. In the early years of 3D printing, rapid design and model manufacturing were primarily used for rapid prototyping, as they significantly accelerated the product development phase.

Currently, 3D/4D printing has broad and versatile industrial applications from the production of single parts or small batches, which fit perfectly into flexible manufacturing systems, to large-scale product series currently manufactured using large-scale 3D printers. Among the desired features in the development of 3D/4D printing technology, the most frequently mentioned are the abilities to

− Customize the product to individual customer needs;
− Design and manufacture components with complex shapes that cannot be manufactured using conventional techniques or are difficult to manufacture using conventional techniques;
− replace several components with a single element;
− Manufacture complex internal structures, e.g., parts within parts; thin-walled components; and spongy, cellular, or honeycomb structures, while maintaining adequate strength.

Three-/four-dimensional printing is an interdisciplinary technology. Both fundamental sciences, such as physics and chemistry, and applied sciences, such as mechatronics, materials science, and automation, are used to develop new technologies and modernize existing ones, produce new materials for 3D/4D printing, and build and operate 3D/4D printers.

In addition to the various (and increasingly advanced) design and technological functions of modern printers and engineering materials, the manufacturing space has been expanding for over a decade to include an additional dimension. This dimension is time. Three-dimensional printing is performed in three-dimensional, static Euclidean space. If the change in material properties over time under the influence of external stimuli is taken into account, a fourth dimension can be considered. The printing process, which incorporates dynamic material changes, is called 4D printing. It can therefore be considered a combination of 3D printing with materials with dynamic properties, e.g., geometric properties. Such materials include, for example, shape memory materials.

When discussing engineering applications in 3D/4D printing, all of the above-mentioned areas must be considered. However, providing a concise and comprehensive overview of all engineering applications and their development prospects is impossible. Therefore, this Special Issue entitled “3D & 4D Printing—in Engineering Applications” may inspire us to present only some of the areas of 3D/4D printing technology and its engineering applications. The following areas can be mentioned:

− Designing and producing molds using laser powder bed fusion (LPBF) technology [1,2,3,4,5,6,7,8,9,10,11,12,13];
− Testing and selecting technological parameters of selective laser melting (SLM) [14,15,16,17,18,19,20,21,22,23,24,25,26,27,28,29,30,31,32,33];
− Researching the properties of materials used in 4D technology [34,35,36,37,38,39,40,41,42];
− Addressing problems of manufacturing thin-walled models [43,44,45,46,47,48,49,50,51];
− Producing and using lattice and cellular structures obtained with 3D printing technology [52,53,54,55,56,57,58,59,60,61,62];
− Examining the dynamic mechanical properties of materials produced using 3D printing technologies [24,25,63,64,65,66,67].

Three-dimensional printing technologies, especially laser powder bed fusion, have proven to be suitable for producing injection molds due to their ability to create various complex shapes, especially conformal cooling channels. Mold cooling enables temperature management and increases mold durability, for example, in the aluminum injection molding process [1]. The authors describes the results of tests on dimensional stability, mechanical properties, and wear resistance under conditions simulating industrial production, comparing inserts made of grade 300 maraging steel with conventional H13 tool steel. The results confirm that additive manufacturing, when combined with customized surface treatment and an optimized cooling system, increases mold durability by up to 2.6 times compared with that of conventional solutions.

A review article [7] extensively discusses cooling channel systems replacing conventional straight-hole cooling systems. The current cooling systems are divided into eight types. The basic type is suitable for simple-shaped parts. More complex types and hybrid molds with straight holes and conformal channels are suitable for complex-shaped parts. Hybrid manufacturing (LPBF–CNC milling) is a future direction for manufacturing molds with conformal channels with high dimensional accuracy.

Selecting technological parameters for additive manufacturing is another area of research focus for various engineering applications. A key process parameter in selective laser melting (SLM) is the degree of alloy path overlap [14], which has a significant impact on the forming quality of, for example, Ti-6Al-4V alloy. In the study, the influence of different degrees of alloy path overlap on the final product quality was considered through a combination of simulation and experiments. The simulation and experimental results showed that excessive alloy path overlap leads to grain growth in the overlap zone and reduces the tensile properties of samples. Therefore, both excessive and insufficient overlap negatively affected the density and mechanical properties of the tested samples.

Another important technological issue in 3D printing is the impact of part orientation on the build platform on mechanical properties. In SLM technology, the anisotropy of mechanical properties can be reduced through appropriate heat treatment after the first stage of part building, as exemplified by 17-4 PH stainless steel [30]. Laser-melted samples exhibited mechanical anisotropy, with the yield strength of a 45° melted sample (976 MPa) being 23.7% higher than that of a 0° melted sample (789 MPa). Heat treatment effectively homogenized the microstructure, eliminating the initial anisotropy and increasing the average yield strength by up to 54% to 1208 MPa. These results show engineers that various forms of heat treatment (depending on the type of material/powder being melted) should be considered after the laser melting process in SLM to improve mechanical properties.

Materials play a key role in 4D printing, which is why most engineering research and applications have focused on this area. An article [34] describes the pyrolytic expansion of polyurethane during the ceramization step, which ensures the deformability and mechanical properties of the resulting ceramic parts. Shape memory materials are relatively common in 4D printing. These include various types of polymers. Shape programming steps typically require stringent temperature requirements (≥90 °C). Remote control is also difficult, seriously limiting the use of polymers [37]. The authors proposed a new thermoplastic polymer system with self-healing and highly stretchable shape memory capabilities for 4D printing based on digital light processing (DLP). This material system is created by integrating two distinct compositions: a polymer-based framework that acts as a reinforcing phase and a flexible, hydrogen-bonded lubricant that facilitates self-healing, high stretchability, and improved shape recovery.

Four-dimensional printing has biomedical applications. An example is the use of scaffolds for cartilage and bone tissue regeneration, which require the appropriate selection of materials and manufacturing techniques [40]. Implant materials must meet various biological requirements: they must be biocompatible, biodegradable, and have appropriate mechanical properties. In the study, the structural, mechanical, thermal, and functional properties of a shape memory terpolymer modified with β-tricalcium phosphate (β-TCP) were evaluated. The developed materials were used in 4D printing for the production of medical implants.

Thin-walled elements are among the more challenging components produced using 3D/4D printing technology. Generally, the minimum wall thickness of a printed element constitutes the lower limit of a given printing technology. A paper [44] presents the results of tests to determine selected quality characteristics of sample models manufactured using 3D printing technology from the powder bed fusion (PBF) group and an aluminum powder-based material. Two characteristics were analyzed: tensile strength and geometric surface structure. Strength tests of thin-walled models were conducted for samples with four specified thicknesses: 1, 1.4, 1.8, and 2 mm, and four printing orientations. The test results showed that thin-walled models had certain technological limitations regarding the minimum sample thickness in the manufacturing process. The strength of the thin-walled models, relative to the reference samples that were called “solid,” depended on sample thickness and printing direction. Roughness parameters were quantified, which determine such functional quality characteristics as friction and wear. The results have applications in the production of specific industrial components.

In [48], the authors found that calculating the energy density alone is insufficient because parts manufactured using similar energy densities but different parameter combinations can have widely different properties and dimensions. Thin-walled parts are particularly susceptible to the influence of processing parameters. The authors of the paper analyzed the effect of laser power and scanning speed on the dimensions of thin-walled Ti6Al4V tubes. Three models were obtained for the wall melt zone thickness, the total wall thickness, and the hole width.

Three-/four-dimensional printing is indispensable in the construction of lattice and cellular structures. Lattice structures, composed of interconnected struts, are effective in reducing the mass of manufactured components while maintaining the required mechanical properties. Due to this potential, the authors of [52] investigated and developed an effective variant of the BCC (body-centered cubic) lattice structure to increase structural strength and energy absorption capacity based on Maxwell’s stability criterion. Electron beam melting (EBM) metal additive technology was used to produce the tested structures. Extensive testing demonstrated that the newly designed lattice structure offers substantial strength benefits, with energy absorption capacity higher by 365% compared with that of existing structures, achieved by incorporating vertical and horizontal lateral support into the original BCC lattice configuration. The improved lattice structures can be used in applications requiring low weight and high strength, such as in the aerospace, marine, and other industries.

In [58], the authors argue that additive manufacturing of polymer ceramics (PDCs) is a breakthrough manufacturing process that incorporates several technologies, such as light curing and inkjet printing. A modified fused deposition model (FDM) was also used to create ceramic structures. Three-dimensional-printed polylactic acid (PLA) honeycomb networks were dip-coated with two preceramic polymers (polyvinylsilazane and allylhydridepolycarbosilane) and then pyrolytically transformed into SiCN and SiC ceramics, respectively. This technology yielded cellular ceramic structures.

Research into the dynamic properties of materials produced via 3D/4D printing is also worth mentioning. Relatively few studies have been published in this area, but their frequency has recently been increasing due to the increasing range of industrially produced components. A work [24] focused on the fatigue tests of Ti6Al4V powder samples produced using SLM technology with horizontal and vertical structures, which were heat-treated after laser sintering. The mechanical properties and failure mechanism were determined using fracture analysis. The critical factors influencing mechanical properties were also assessed, as well as the correlation between fatigue life and damage sources. The results show that the mechanical properties were determined by the morphology of the α phase and defects, which included micropores and a lack of fusion defects. The results of such studies are important for improving engineering applications involving 3D printing technologies.

## 2. Standardization in Engineering Aspects

Whenever we discuss industrial 3D/4D printing and engineering applications, standardization issues must not be overlooked. Standards are required in implementing industrial procedures.

Currently, the two largest international standards organizations, ISO and ASTM, are engaged in advanced work on developing new standards to streamline engineers’ work. Both organizations are collaborating on 3D printing standards, creating ISO/ASTM standards. Below, we present a description of selected 3D printing standards with the potential for application by engineers working with layered manufacturing.

The first, fundamental standard that a 3D printing engineer should use is **ISO/ASTM 52900:2021 Additive manufacturing—General principles—Fundamentals and vocabulary** [68]. This standard contains information on the vocabulary used in 3D printing and serves as a ***language of communication*** for the manufacturing and ordering of 3D printing, 3D printer servicing, and research in this area. This 2021 standard regulates new technology names, introducing, among others, the name MEX (material extrusion) instead of the well-known names FDM (fused deposition modeling) and FFF (fused filament fabrication).

Another important standard for engineers is **ISO/ASTM 52901:2017 Additive Manufacturing—General Principles—Requirements for Purchased AM Parts** [69]. In engineering applications, the vocabulary described above is crucial but equally important is establishing the scope of work related to 3D printing and its testing methods. The standard specifies which issues should be included in the contract for 3D printing of components and which acceptance, testing, and repair methods should be omitted.

The final standard presented with practical engineering significance is **ISO/ASTM 52926-1:2023 Additive manufacturing of metals—Qualification principles, Part 1: General qualification of operators** [70]. This standard provides guidelines for the training, qualifications, and essential knowledge of 3D printing worker/engineer. This standard is crucial in the field of metal 3D printing due to the complex processes of 3D printing technology, heat treatment, machining, and maintenance of 3D printing machines.

## 3. Conclusions

To summarize this short analysis, the development of 3D/4D printing technology requires the use of increasingly advanced engineering applications in many areas of science, especially applied sciences.

Furthermore, the international standards relevant to the work of engineers and 3D printing technology are essential. In the future, these standards will be widely adopted as a prerequisite for working with advanced 3D printing systems. This approach will contribute to increases in the quality of models produced via 3D printing.

This Special Issue covering 3D and 4D printing in engineering applications encompasses many areas, both scientific and specifically engineering, that require development.
